# IFN-γ induced the formation of foamy macrophages via CD40 signal to control *Mycobacterium abscessus* pulmonary infection

**DOI:** 10.3389/fimmu.2025.1697443

**Published:** 2025-11-27

**Authors:** Shaoyan Zhang, Ya Feng, Xianwei Wu, Jiajun Chen, Huiyong Zhang, Li Tian, Hai Lou, Liping Wang, Ben Su, Xing Huang, Lei Qiu, Dingzhong Wu, Wei Sha, Zhenhui Lu

**Affiliations:** 1 Institute of Respiratory Diseases, Longhua Hospital, Shanghai University of Traditional Chinese Medicine, Shanghai, China; 2 Department of Tuberculosis, Shanghai Pulmonary Hospital, Tongji University School of Medicine, Shanghai, China

**Keywords:** *Mycobacterium abscessus*, foamy macrophages, lipid droplets, IFN-γ, CD40

## Abstract

**Background:**

Foamy macrophages (FMs) have recently shown potential in restricting the intracellular growth of *Mycobacterium.* However, the mechanism behind the formation of FMs and their significance in the pathophysiology of *Mycobacterium abscessus* (*M. abscessus*)-induced pulmonary infections remains poorly understood.

**Methods:**

Clinical blood samples, murine infected models (WT and IFN-γ^-/-^ mice), and macrophage infected models were utilized to investigate the formation of FMs mediated by IFN-γ and its critical role in bacterial control during *M. abscessus* infection. Oil Red O staining and confocal microscopy were employed to assess the effect of IFN-γ on FMs formation *in vivo* and *in vitro*, coupled with specific signaling pathway inhibitors. Transcriptomics and lipidomics were performed to identify key pathways, genes, and lipid metabolites.

**Results:**

During the acute infection phase, the lipid droplets (LDs) in peripheral blood mononuclear cells significantly increased, along with the upregulation of serum IFN-γ levels. Experiments with IFN-γ^-/-^ mice infected with *M. abscessus* revealed that IFN-γ is essential for the formation of LDs or FMs during the infection. The addition of IFN-γ increased the formation of LDs or FMs and restricted the growth of *M. abscessus in vitro* and *in vivo*. Furthermore, we found that IFN-γ induced the formation of LDs required CD40-DGAT1 signaling, and a significant positive correlation between serum IFN-γ and sCD40 levels was observed. Lipidomics analysis revealed significant metabolic reprogramming in FMs, with triacylglycerols (TAGs) identified as the most significantly altered lipid species. Notably, TAGs containing fatty acid side chains such as linoleic acid and palmitic acid may play crucial roles in host defense during infection.

**Conclusion:**

This study established the IFN-γ-CD40-DGAT1 axis as an important role in the formation of FMs and the control of *M. abscessus* infection. These findings revealed a critical immune-metabolic pathway that may be leveraged for host-directed therapeutic interventions.

## Highlights

- Exogenous IFN-γ supplementation enhanced LDs/FMs formation and inhibited *M. abscessus* growth both *in vitro* and *in vivo*.
- IFN-γ induces lipid metabolic reprogramming by activating the CD40-DGAT1 pathway.
- Multi-omics analysis characterized the molecular and metabolic profiles of IFN-γ-induced FMs in controlling *M. abscessus* infection.

## 1 Introduction

*Mycobacterium abscessus* (*M. abscessus*) infects both patients without underlying risk factors and patients with a history of pulmonary disorders, including cystic fibrosis and bronchiectasis. It was the first rapidly growing mycobacteria to be isolated (1). Typically, *M. abscessus* is resistant to most chemotherapies, making it impossible to treat the infection despite prolonged combination antibiotic therapy (2), and leading to an accelerated decline in lung function (3, 4). Moreover, using whole genome sequencing of a global collection of clinical isolates, it was found that the majority of *M. abscessus* infections were acquired through transmission, potentially via fomites and aerosols, of recently emerged dominant circulating clones that have spread globally (5). Furthermore, the treatment success rate across *M. abscessus* pulmonary disease (MAB-PD) was 45.6% (6), and the overall 5-, 10-, and 15-year cumulative mortality rates were 11.4%, 29.8%, and 50.6%, respectively (7). Thus, identifying new or repurposed drugs are of utmost importance. However, the pathological changes involved and host defense mechanisms of *M. abscessus* infections are still unclear.

Foamy macrophages (FMs), specialized macrophages with a foamy appearance, are distinguished by their numerous lipid droplets (LDs) and vesicles (8). During *Mycobacterium tuberculosis* (*M.tb*) infection, these FMs serve a dual role. On one hand, the cytoplasmic accumulation of fatty acids (FAs) enhances the inflammatory potential of FMs and impedes the intracellular growth of *M.tb* (9–11). On the other hand, *M.tb* actively encourages lipid accumulation, using these lipids as energy and carbon sources (12, 13). This process drives the differentiation of infected macrophages into FMs, ultimately forming granulomatous lesions centered on FMs and surrounded by lymphocytes, which sustain chronic *M.tb*-induced pulmonary inflammation and systemic infections (14). In chronic infections caused by *M. abscessus*, granulomas are also produced, just as in *M.tb* infections. A previous study, which utilized a zebrafish model to evaluate *M. abscessus*-induced granuloma formation, revealed the role of TNF in activating macrophage and neutrophil recruitment and granuloma formation (15). As an important inflammatory cytokine, IFN-γ and its associated effector molecules have been extensively studied in the field of anti-infection (16, 17). Studies have found that LDs formation in *M.tb*-infected macrophages requires IFN-γ/HIF-1α signaling and supports host defense (18). However, the formation of FMs in *M. abscessus-*induced pulmonary infections has not yet been reported.

CD40, a critical macrophage surface antigen, plays essential immunomodulatory functions in cardiovascular diseases and microbial infections (19, 20). Current research indicates that the activation of CD40 is closely associated with the M1-type polarization of macrophages (21, 22). M1 macrophages release pro-inflammatory cytokines and produce protective responses that lead to antimicrobial or antitumor activity (23–25). In atherosclerosis, macrophage-specific CD40 deficiency attenuates pro-inflammatory phenotypic switching and intracellular lipid accumulation (26). Although CD40 has been established as a potent host-directed therapeutic target against *M. tb*, regulating key processes such as autophagy (27), dendritic cell-mediated antigen presentation (28), and T-cell immunity (29), its connection to the biology of FMs remains unexplored.

In this study, we found that the formation of FMs were required during acute *M.abscessus* pulmonary infection *in vitro* and *in vivo*. Moreover, experiments with IFN-γ^-/-^ infected mice revealed that IFN-γ is essential for the development of LDs or FMs during *M. abscessus* infection. Consequently, the administration of IFN-γ presents a potential strategy to enhance FM formation and inhibit *M. abscessus* growth, both *in vitro* and *in vivo*. We also found that IFN-γ induces the formation of LDs, which requires the CD40-DGAT1 signal to trigger lipid metabolic reprogramming. The levels of IFN-γ and sCD40 in the serum of acute MAB-PD patients were significantly elevated and exhibited a positive correlation. In summary, our findings demonstrate that the IFN-γ-CD40-DGAT1 axis plays an important role in the formation of FMs and in controlling *M. abscessus* infection.

## 2 Methods

### 2.1 Human serum and PBMCs samples

All subjects provided written informed consent, and the ethics was approved by Shanghai Pulmonary Hospital (NO. K24-702) and Longhua Hospital affiliated with Shanghai University of Traditional Chinese Medicine (NO. 2024LCSY166). Human serum and peripheral blood mononuclear cells (PBMCs) were obtained from patients with MAB-PD. The PBMCs were isolated using Ficoll-Paque™ PLUS (Cytiva, Wilmington, USA) density gradient centrifugation, in accordance with the manufacturer’s instructions. Based on clinical signs, blood tests, and chest imaging examinations, the clinical stage of each patient was classified at each visit as either stable disease (defined by the absence of fever and no signs of active infection) or active disease (characterized by fever and signs of active infection as determined by blood tests or chest imaging, or requiring hospitalization). Patients with two or more non-tuberculous mycobacteria infections, or those with active pulmonary tuberculosis, were excluded.

### 2.2 Bacterial strains and culture conditions

*M. abscessus* clinical isolate 715 (rough colony variants) was a kind gift from Professor Fangyou Yu from Shanghai Pulmonary Hospital (30). pMV361-mcherry *M. abscessus* 715 was developed by Gene-optimal (Shanghai, China). To summarize, the mCherry gene was inserted into the pMV361 plasmid using the EcoRI and HindIII restriction sites. The correctly integrated plasmid was then electroporated into *M. abscessus* 715 using a voltage of 2.5 kV and a capacitance of 25 μF. Positive clones were screened and identified using kanamycin-resistant plates at a concentration of 250 μg/mL. The successfully identified single clones were stored at -80°C. *M. abscessus* 715 was grown in liquid Middlebrook 7H9 broth or solid medium Middlebrook 7H10 agar at 37°C as previously described (31). Bacterial cultures were grown in Middlebrook 7H9 medium at 37°C under constant agitation (200 rpm) and harvested after 3 days during the mid-logarithmic phase. To prepare fresh bacterial broth, the bacterial precipitate was harvested through centrifugation at 4,000g for 5 minutes and subsequently washed twice with sterile phosphate-buffered saline (PBS). The bacterial culture was then resuspended in sterile PBS. Turbidity was quantified in McFarland units (MCF) using a Turbidimeter (bioMérieux S.A., Marcy l’Etoile, FR). Plate counting was performed on bacterial suspensions with varying MCFs, and a conversion factor specific to our experimental strains was established: 1 MCF is approximately equivalent to 2.9×10^8^ CFU/mL.

### 2.3 Cell culture

Primary mouse bone marrow-derived macrophages (BMDMs) were generated from the bone marrow of 6-8-week-old male C57BL/6 mice as previously described (32). Cells were grown for 5–6 days in RPMI 1640 medium supplemented with 1% non-essential amino acids (Sigma, Darmstadt, Germany), 20 ng/mL M-CSF (Novoprotein, Jiangsu, China), 10% fetal bovine serum (South Logan, UT, USA), and 1% penicillin–streptomycin (Gibco, CA, USA).

### 2.4 Mice

Female wildtype C57BL/6J mice (aged 6–8 weeks) were obtained from Charles River Laboratory Animal Technology Co. Ltd. (Zhejiang, China). IFN-γ^-/-^ mice on a C57BL/6 background were purchased from Cyagen (Suzhou, China). Animal experiments were approved by the Institutional Animals Care and Use Committee (IACUC) of Shanghai Institute of Immunity and Infection, Chinese Academy of Sciences (Approval No. P2021024).

### 2.5 Mouse intratracheal infection model

At the time of inoculation, WT (n=5–7 per time point) or IFN-γ^-/-^ mice (n=4–6 per time point) were anesthetized using 80 mg/kg body weight ketamine and 10 mg/kg body weight xylazine and were infected via intratracheal delivery with *M. abscessus* at 5×10^7^ CFU in 40 μL of sterile PBS once (33). The blank group received tracheal inhalation of sterile PBS.

For IFN-γ treatment, WT mice (n=5 per group) were administrated with 50 ng recombinant IFN-γ (novoprotein, Jiangsu, China) via intranasal inoculation one day before intratracheal infection, and treatment was continued on days 1 and 3 post-infection (34). At indicated days post-infection, mice were sacrificed, and lung tissue was harvested in a sterile fashion.

### 2.6 Histological analysis and Oil Red O staining

After the lung is removed from the body, an adequate amount of 10% neutral buffered formalin solution is gently injected into the lung through the main bronchus to restore the lung tissue to an expansion degree similar to its physiological state. The lung is then completely immersed in formalin for fixation. Following thorough fixation, experienced pathologists meticulously conduct a gross examination of the specimens and perform systematic sectioning and sampling in accordance with standard operating procedures. Thin slices (4 μm) were stained with Hematoxylin & Eosin staining (H&E staining). To demonstrate the lipids, cryosections were stained with Oil Red O, dissolved in isopropyl alcohol, and counterstained with hematoxylin.

### 2.7 Enzyme-linked immunosorbent assay

In accordance with R. González-Tajuelo’s methodology (35), the right middle lung was mechanically disrupted in 1×PBS. After four freeze - thaw cycles were performed to disrupt the cell membranes, the samples were centrifuged at 5,000 g for 5 minutes at 4°C, and the supernatants were collected for IFN-γ detection. The concentration of IFN-γ in lung tissues and cell culture supernatant was determined using an IFN-γ ELISA kit, in strict accordance with the manufacturer’s instructions (Multisciences, Zhejiang, China). Although histological and ELISA samples were derived from two distinct batches of animal experiments, the murine *M. abscessus* infection model exhibited stability, as corroborated by the lung bacterial load. The serum levels of IFN-γ and sCD40L were quantified using an IFN-γ and sCD40L ELISA Assay kits, respectively (Multisciences, Zhejiang, China). The serum level of sCD40 was determined using a sCD40 ELISA Assay kit, adhering to the manufacturer’s protocol (Epizyme Biotech, Shanghai, China). Additionally, the serum levels of triacylglycerol (TAG) and total cholesterol (T-CHO) were assessed using specific kits, as per the instructions provided (Nanjing Jiancheng Bioengineering Institute, Jiangsu, China).

### 2.8 Flow cytometric analysis

To detect FMs in the blood, the following method was applied. Human-PBMCs were stained with the following antibodies after the separation: Dye eFluor™ 780, APC-labeled anti-CD14 and BODIPY 493/503. Mice-PBMCs were stained with the following antibodies: Dye eFluor™ 780, BV510-labeled anti-CD45, PC5.5-labeled anti-F4/80 and BODIPY 493/503.

To detect the immune cell typing in the infected lung tissue, the right lower lobe of lung tissue was minced and digested with 50 μg/ml Liberase™ (Roche, Basel, Switzerland) and 1 μg/mL DNase I (Sigma, St. Louis, MO, USA) for 45 min at 37 °C. After obtaining a cell suspension, the cells were stained with the following antibodies. For the myeloid cells, cells stained with Dye eFluor™ 780, BV510-labeled anti-CD45, FITC-labeled anti-CD11b, PE-labeled anti-CD11c, AF700-labeled anti-Ly-6G, BV421-labeled anti-F4/80 and PE/Cyanine7-labeled anti-MHC II. FMs were defined as CD11c^high^MHC II^high^ population (36). For the lymphocytes, cells stained with Dye eFluor™ 780, BV510-labeled anti-CD45, PE/Cyanine7-labeled anti-CD3, BV421-labeled anti-CD4, PE/Cyanine5.5-labeled anti-CD8, BV605-labeled anti-NK1.1 and PE-labeled anti-IFN-γ. Analyses were performed with an acquisition of 30,000 live events.

Flow cytometry was performed using Beckman CytoFlex LX (Beckman, Miami, FL, USA) with CytExpert Software version 2.0.

### 2.9 Confocal microscopy analysis

For confocal imaging of PBMCs, isolated PBMCs were resuspended in RPMI-1640 medium with 10% fetal bovine serum and 1% penicillin–streptomycin, seeded in a 24-well glass bottom plate (Cellvis, Mountain View, CA, USA) at 1×10^6^ cells/mL, and incubated at 37 °C with 5% CO_2_. After 4 h, non-adherent cells were washed away with PBS, and adherent cells were fixed with 4% formalin for subsequent confocal imaging.

For confocal imaging of BMDMs, cells were plated on 24-well glass bottom plates at a density of 5×10^5 cells per well. BMDMs were pretreated with 6.25 ng/mL of recombinant murine IFN-γ (Novoprotein, Jiangsu, China). Following overnight pretreatment with IFN-γ, BMDMs were infected with *M. abscessus* at a MOI of 10. After a 2-hour phagocytosis period, the media was replaced, and IFN-γ was added again at 6.25 ng/ml to all IFN-γ pretreated wells. Infected cells were treated with chemical inhibitors or vehicle controls at the time of infection. Inhibitor doses were selected based on relevant references. The following inhibitor concentrations were used: 10 μM Ruxolitinib (37), 100 μM DRI-C21045 (a CD40-CD40L costimulatory protein-protein interaction inhibitor) (38), 10 μM T863 (DGAT1 inhibitor) (39). All inhibitors were purchased from MedChemExpress LLC (MCE, New Jersey, USA). At 24 h post-infection, the supernatant was discarded and the cells were washed twice with sterile PBS. Cells were fixed in 4% formalin for 1 h, washed with PBS, permeabilized with 0.1% Triton X-100 for 10 min, and stained with DAPI and BODIPY 493/503 (Thermo Fisher Scientific, Waltham, MA, USA) each at a concentration of 1 μg/ml in PBS for 1 h, followed by washing with PBS three times. Images were captured on a Leica MICA confocal microscope at 63× magnification.

### 2.10 Transcriptome sequencing analysis

Total RNA was extracted from BMDMs using TRIzol (Invitrogen, CA, USA) according to manual instruction. Subsequently, total RNA was qualified and quantified using a Fragment Analyzer or Agilent 2100 Bioanalyzer (Agilent, CA, USA). RNA samples were sequenced using DNBSEQ-T7 at the Wuhan BGI Technology Service Co., Ltd. Almost 4–5 biological replicates were performed per condition. Quality-filtered RNA-seq reads were aligned to the mouse genome (GCF_000001635. 27_GRCm39) by Bowtie2 software. In our study, differentially expressed genes (DEGs) were filtered with 2-fold cut-off and adj *p* < 0.05.

### 2.11 Intracellular CFU assay

For the CFU assay, BMDMs were pre-treated with IFN-γ (6.25 ng/mL) or for 12 h, then infected with *M. abscessus* (MOI = 10) for 2 h and treated with amikacin (200 μg/mL) to clear extracellular bacteria. Infected cells were treated with inhibitors (100 μM DRI-C21045 or 10 μM T863), continuously stimulated with IFN-γ, and lysed 24 h post-infection for CFU quantification on 7H10 agar plates.

### 2.12 FAs-mediated formation of FMs

BMDMs were pre-treated with 500 μM oleic acid/palmitic acid cocktail (2:1 ratio) (112-80-1; 57-10-3, St. Louis, USA) for 12 h prior to infection. Following the established infection protocol, intracellular lipid content and bacterial load were assessed 24 h post-infection.

### 2.13 Measuring levels of TAG and T-CHO

The lung tissues were weighed accurately and added to 9 times the volume of phosphate buffer at a ratio of weight (g): volume (mL) = 1:9. Tissues were homogenized mechanically in an ice-water bath, then centrifuged at 2500 rpm at 4°C for 10 min, and the supernatant was collected for measurements. The protein concentration in each sample was determined according to the instructions of the BCA detection kit (Beyotime Biotechnology, Shanghai, China). The lung tissues and serum levels of TAG and T-CHO were measured according to the instructions of specific kits (Nanjing Jiancheng Bioengineering Institute, Jiangsu, China). The absorbance of each well was measured at 500 nm using a Microplate reader (TECAN, Switzerland).

### 2.14 Lipidomics and analysis

BMDMs (1×10^7^ cells per sample) were harvested, and cellular reactions were immediately quenched by flash-freezing in liquid nitrogen. Samples were thawed on ice to minimize degradation. Each cell pellet was resuspended in 10 μL deionized water, homogenized for 3 min with 10 grinding beads, and subsequently extracted with 300 μL lipid extraction solvent (MSC-100, Allsheng Instruments Co., Ltd., Hangzhou, China) during an additional 3-min homogenization step. Following homogenization, samples were vortex-mixed at 1,200 rpm for 20 min at 10°C and centrifuged at 4,000 ×g for 20 min at 4°C. A 20 μL aliquot of the supernatant was transferred to a 96-well plate and mixed with 80 μL methanol containing 5 mM ammonium acetate for LC-MS analysis. Targeted lipidomics was performed using ultra-performance liquid chromatography coupled with triple quadrupole mass spectrometry (UPLC-TQMS; ACQUITY UPLC-Xevo TQ-S, Waters Corp., Milford, MA, USA). Raw data were processed with MassLynx software (v4.1, Waters) for peak extraction, integration, and quantification of individual lipids. Subsequent statistical analyses were conducted using iMAP software (v1.0, Metabo-Profile, Shanghai, China). In our study, differentially expressed metabolites were filtered with 1.5-fold cut-off and *p* < 0.05.

### 2.15 Quantitative real-time PCR

Total RNA was extracted from BMDMs samples using Trizol reagent, and complementary DNA was synthesized with the PrimeScript RT Master Mix (Perfect Real Time) (TaKaRa, Tokyo, Japan). Real-time PCR (RT-PCR) was performed using the ChamQ Universal SYBR qPCR Master Mix (Vazyme, Jiangsu, China).

The following primers were used:

CD36 forward: 5’-ATGGGCTGTGATCGGAACTG-3’, reverse: 5’- GTCTTCCCAATAAGCATGTCTCC-3’.
CD40, forward: 5’-TCACCATTTTCGGGGTGTTTC-3’, reverse: 5’- CCGCAGGGGGTAACATCTC-3’.
IL-12β, forward: 5’-TGGTTTGCCATCGTTTTGCTG-3’, reverse: 5’- ACAGGTGAGGTTCACTGTTTCT-3’.
DGAT1, forward: 5’-TCCGTCCAGGGTGGTAGTG-3’, reverse: 5’- TGAACAAAGAATCTTGCAGACGA-3’.
Actin, forward: 5’-GGCTGTATTCCCCTCCATCG-3’, reverse: 5’-CCAGTTGGTAACAATGCCATGT-3’.

### 2.16 Statistical analysis

Student’s t-test or one-way analysis of variance (ANOVA) was employed for statistical analysis and differences between groups were compared. Statistical analysis and graphs were generated using GraphPad Prism 8.0 software (GraphPad Software Inc., San Diego, CA, USA). *p* < 0.05 was considered statistically significant.

## 3 Results

### 3.1 The formation of FMs in *M.abscessus* pulmonary infection

**Figure 1:**
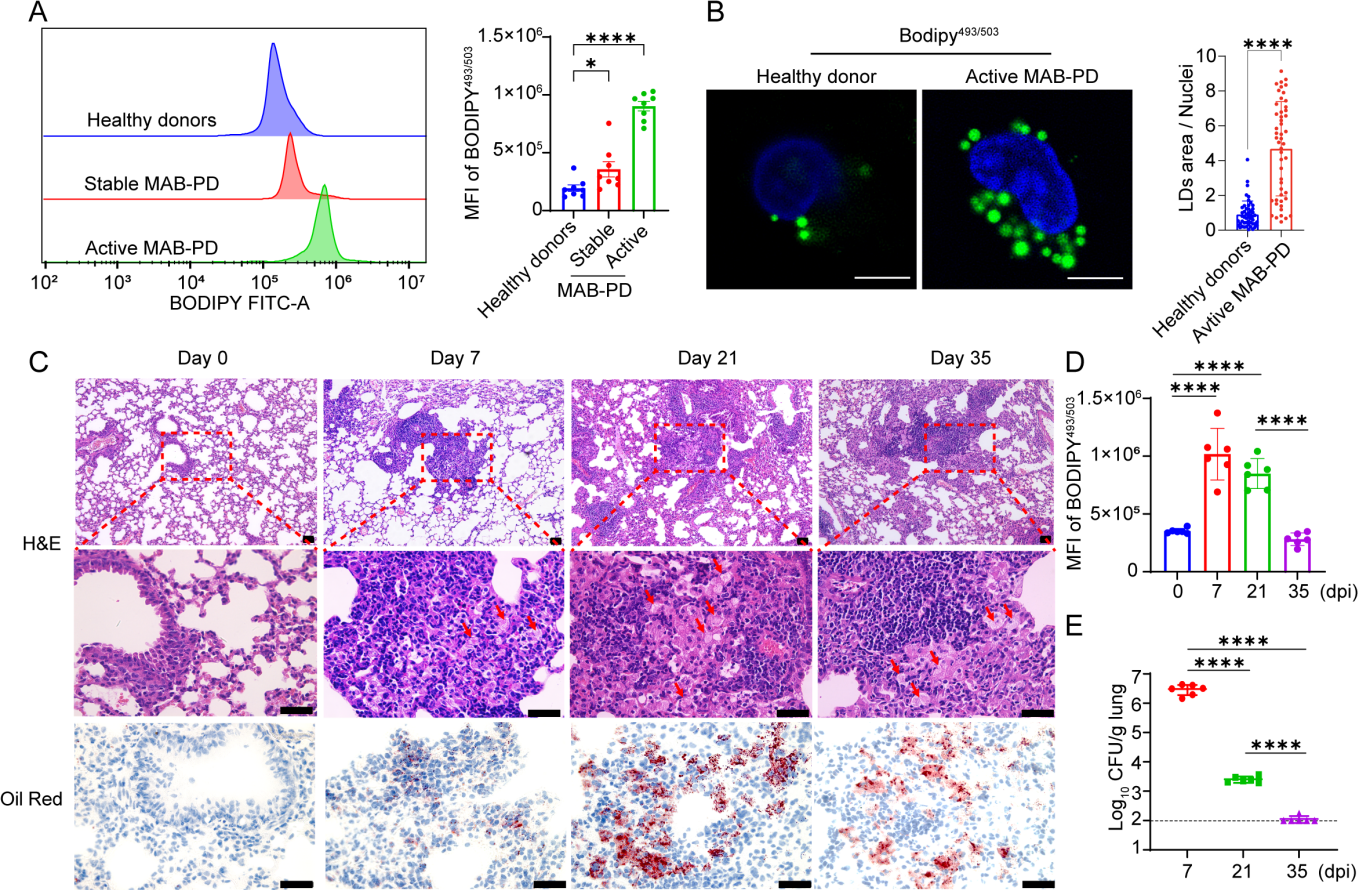
The formation of FMs in *M. abscessus* infection *in vivo* and *in vitro*. **(A)** The mean fluorescence intensity (MFI) of BODIPY493/503 in CD14^+^ monocytes was analyzed by flow cytometry among PBMCs isolated from healthy donors and patients with stable or active *M.abscessus* infection (*n* = 8). **(B)** Lipid staining of monocytes among PBMCs isolated from healthy donors and patients with active *M.abscessus* infection. Scale bar, 10 μm. Quantification of the average size of LDs per PBMC was conducted across 50 microscope fields. **(C)** WT mice were infected via intratracheal delivery with *M. abscessus* at 5×10^7^ CFU under anesthesia and were euthanized on days 7, 21, and 35 dpi. Micrographs of hematoxylin and eosin-stained lung sections prepared at 0 d and on 7-, 21-, and 35 dpi. Staining of neutral lipids in lung cryosections using Oil Red O at 0 d and on 7-, 21-, and 35 dpi. Scale bars, 40 μm. **(D)** The MFI of BODIPY493/503 in CD45+CD11b+ monocytes was analyzed by flow cytometry among blood cells isolated from *M.abscessus* infected WT mice at 0 d and on 7-, 21-, and 35 dpi (n=6). **(E)** Bacterial loads in the lung lysate of *M.abscessus* infected WT mice were determined at 0 d and on 7-, 21-, and 35 dpi (n=6). Data are presented as mean ± SEM and *p* values were determined by one-way ANOVA with Dunnett’s multiple comparison tests. Statistical significance was established at the levels of *p < 0.05 and ****p < 0.0001. MAB-PD, *M.abscessus* pulmonary disease; H&E, hematoxylin-eosin staining.

To investigate the changes in lipid contents during *M.abscessus* infection, we utilized flow cytometry to quantify the levels of human-FMs (CD14^+^BODIPY^+^) among PBMCs in stable and active MAB-PD patients, using healthy donors as controls. The results revealed that the mean fluorescence intensity (MFI) of BODIPY 493/503-a neutral lipid dye-was higher in patients with active MAB-PD than in healthy donors, while stable patients exhibited only a modest increase (**Figure 1A**, **Supplementary Figure S1A**). Furthermore, confocal microscopy analysis of BODIPY 493/503-stained monocytes isolated from healthy donors and active MAB-PD patients confirmed that monocytes from active MAB-PD contained notably more LDs (**Figure 1B**).

To assess changes in the content of LDs in the lungs of mice infected with *M.abscessus*, infected WT mice were euthanized on 7, 21, and 35 dpi (days post infection). H&E staining of lung sections revealed that infected mice developed granulomatous lesions on 7, 21, and 35 dpi, characterized by an aggregation of lymphocytes and FMs (**Figure 1C**). To ascertain the presence of foam-like fatty substances, cryosections were stained with Oil Red O for neutral lipids, revealing that lipids were abundant in WT mice (**Figure 1C**). Concurrently, paralleling the detection of FMs in patient blood samples, we noted that the proportion of mice with FMs (CD11b^+^F4/80^+^BODIPY^+^) in their blood also exhibited a gradual decline with the progression of infection, corresponding to a reduced pulmonary bacterial load (**Figures 1D-E**). In summary, the formation of FMs occurs during acute *M. abscessus* infection.

### 3.2 IFN-γ was required for the formation of FMs in *M.abscessus* infected mice

**Figure 2:**
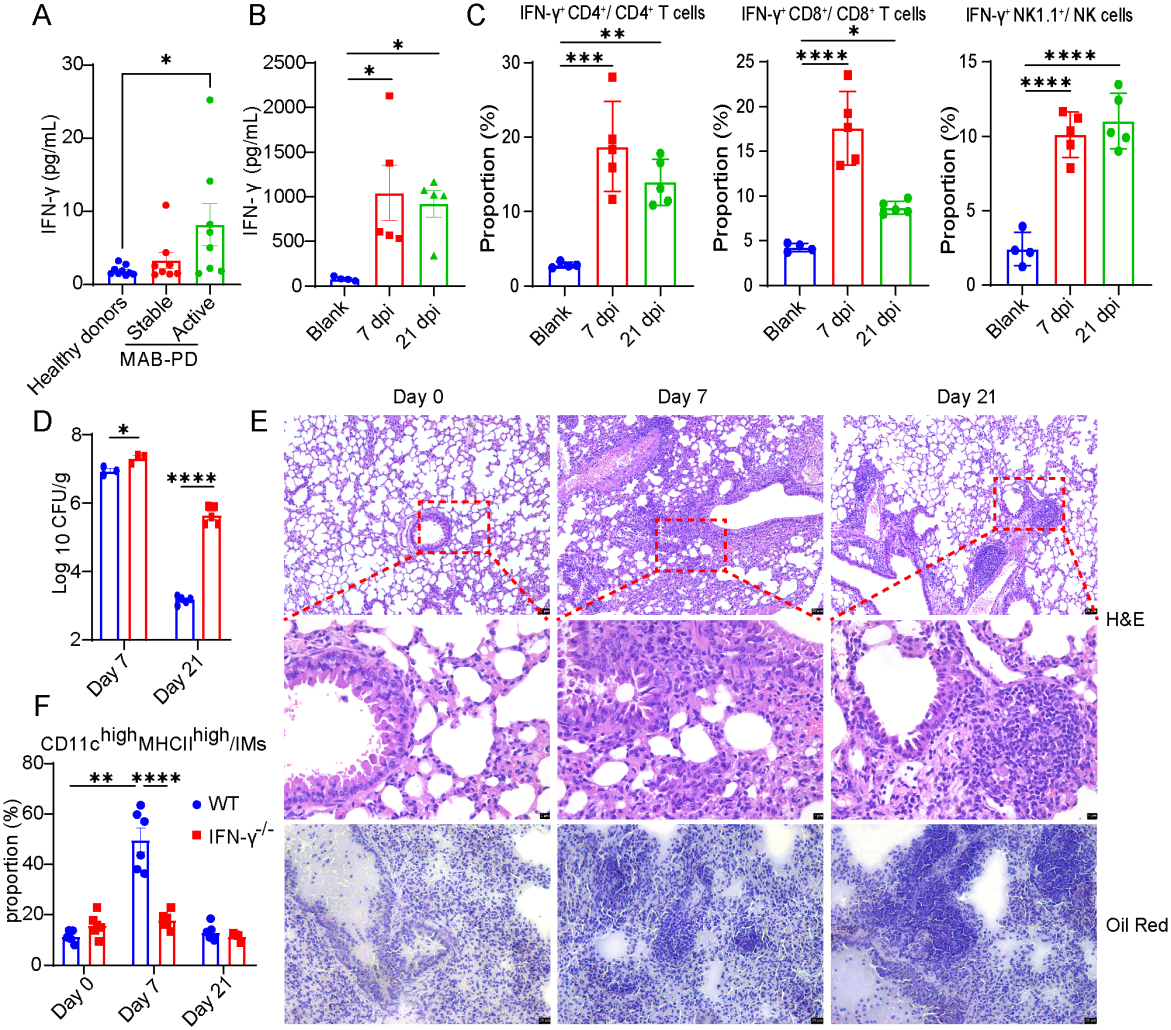
IFN-γ was required for the formation of FMs in *M. abscessus* infected mice. **(A)** Serum IFN-γ concentrations in healthy donors, stable and active MAB-PD patients (*n* = 8-9). **(B)** IFN-γ concentration in lung lysates from mice at 0 d and on 7-, and 21 dpi (*n* = 4-5). **(C)** The percentage of IFN-γ^+^CD4^+^T, IFN-γ^+^CD8^+^T and IFN-γ^+^NK cells in infected mice (*n* = 5), compared to uninfected mice (*n* = 4) on 7-, and 21 dpi using flow cytometry. IFN-γ^-/-^ mice were infected via intratracheal delivery with *M. abscessus* at 5×10^7^ CFU and were euthanized on 7-, and 21 dpi. **(D)** Bacterial loads in the lung lysate of WT and IFN-γ^-/-^ mice were determined on 7-, and 21 dpi (*n* = 3–5 mice per respective time point). **(E)** The percentage of foam interstitial macrophages (FM-IMs) in infected IFN-γ^-/-^ mice (*n* = 4-6), compared to infected WT mice (*n* = 6-7) on 7-, and 21 dpi using flow cytometry. **(F)** Micrographs of H&E and Oil Red O -stained lung sections of IFN-γ^-/-^ mice prepared at 0 d and on 7-, and 21 dpi. Scale bars, 20 μm, 5 μm and 20 μm, respectively. Data are mean ± SEM and *p* values were determined by one-way ANOVA with Dunnett’s multiple comparison tests **(A-C)** or unpaired two-tailed Student’s t-test **(D-E)**. Statistical significance was established at the levels of *p < 0.05, **p < 0.01, ***p < 0.001, and ****p < 0.0001. MAB-PD, *M.abscessus* pulmonary disease; H&E, hematoxylin-eosin staining.

Next, we delved into the mechanism of FMs formation during *M.abscessus* infection. Previously, we observed that lipids were seldom present in Rag2^-/-^ infected mice at 7, 21, and 35 dpi (**Supplementary Figure S2A**). Rag2^-/-^ mice lack mature lymphocytes, and H&E staining revealed extensive pathological damage without the formation of granulomatous lesions (**Supplementary Figure S2B**). Consequently, the aggregation of T lymphocytes to the granulomatous lesions following *M. abscessus* infection may be crucial for FMs formation. Cytokines are essential for FMs formation and the containment of *Mycobacterium* within granulomas (15). IFN-γ/HIF-1α signal has been demonstrated to be required for the formation of LDs during *M. tb* infection (18). We hypothesize that IFN-γ also plays an important role in FMs formation during *M. abscessus* infection. Initially, we measured serum IFN-γ in MAB-PD patients. The IFN-γ levels in patients during a stable phase were comparable to those of healthy individuals. However, acute MAB-PD patients exhibited higher serum IFN-γ levels than those without the condition (**Figure 2A**). Additionally, we assessed IFN-γ levels in the lung tissue of infected mice. At 7 and 21 dpi, IFN-γ levels also notably elevated (**Figure 2B**). Flow cytometry analysis indicated that CD4+T cells, CD8^+^T cells, and NK cells could secrete varying levels of IFN-γ (**Figure 2C**). Furthermore, we investigated the relationship between the increase in IFN-γ levels induced by *M. abscessus* infection and FMs formation.

Subsequently, IFN-γ^-/-^ and WT mice were infected, and the number of bacteria in the lungs was quantified, and the accumulation of FMs in the lungs of infected mice was assessed by means of histological staining and flow cytometry. WT mice showed a robust anti-*M. abscessus* effect. Lung bacteria were counted as 6.9 log_10_CFU and 3.2 log_10_CFU on 7 and 21 dpi, respectively; Compared to infected WT mice, lung bacteria in the IFN-γ^-/-^ group were counted as 7.3 log_10_CFU and 5.6 log_10_CFU on 7dpi and 21 dpi, respectively. The bacterial count in the lungs was consistently higher compared to that in WT mice, and high levels of bacterial colonies were maintained on 21 dpi (**Figure 2D**). H&E staining of lung sections showed that IFN-γ^-/-^ mice also formed granulomatous lesions on 7 and 21 dpi; however, in the area of granulomatous lesions, aggregation of FMs was rarely seen in IFN-γ^-/-^ infected mice on 7 and 21 dpi (**Figure 2E**). Similarly, we found that lipid aggregation was not observed in the lungs of IFN-γ^-/-^ infected mice (**Figure 2E**). This was consistent with the reduced foamy interstitial macrophages (FM-IMs) ratio, which was defined as a group of CD11c^high^MHCII^high^ cells as previously described (36). The proportion of FM-IMs were increased dramatically on 7 dpi (an increase of more than 4 times during infection) in WT infected mice (**Figure 2F**). In summary, IFN-γ is required for the formation of LDs or FMs during an *M. abscessus* infection *in vivo*.

### 3.3 Addition of IFN-γ as a potential pathway to rescue *M. abscessus* infection with increasing formation of FMs

**Figure 3:**
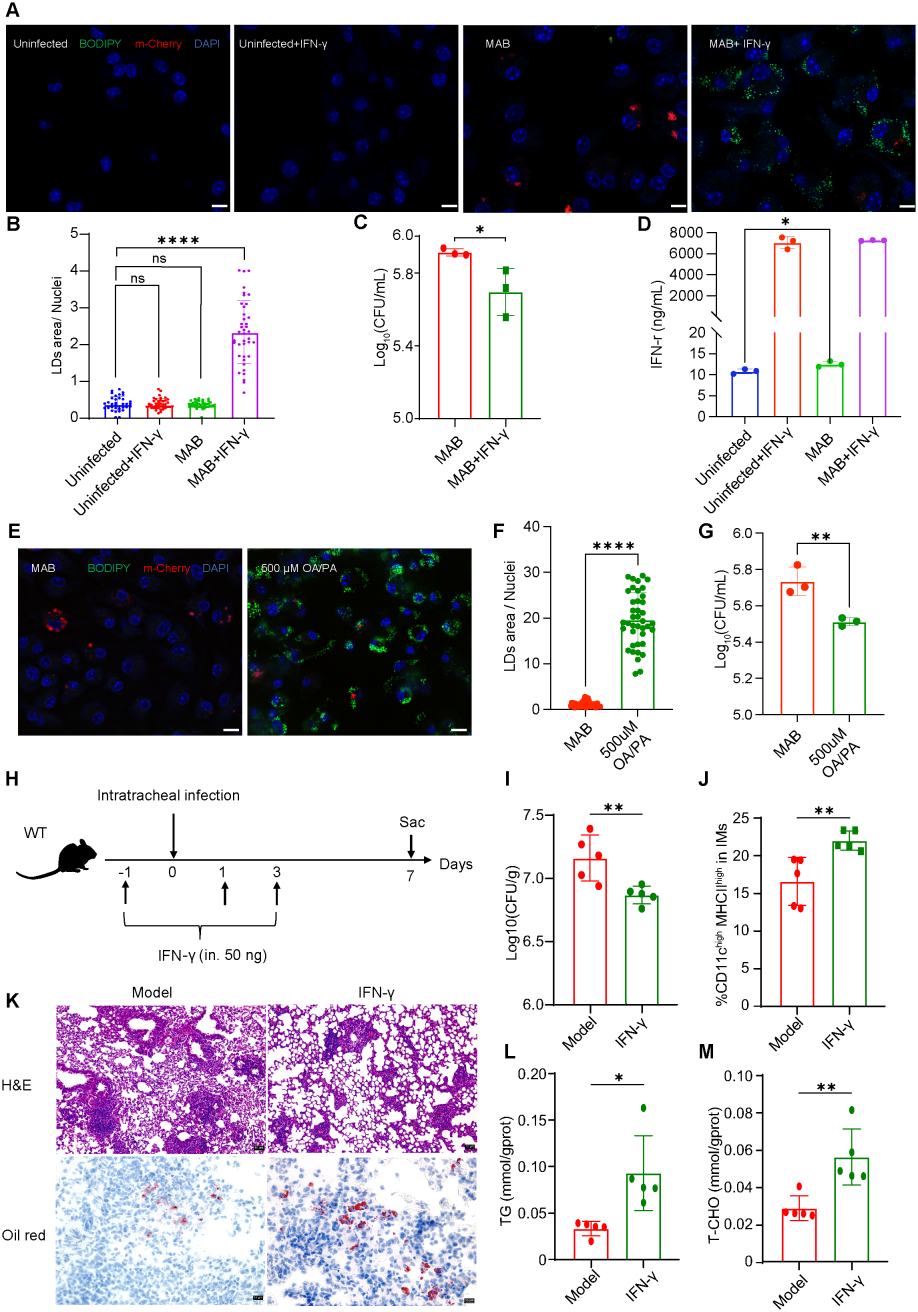
Treatment with IFN-γ increases the formation of FMs and reduces the growth of *M. abscessus*. **(A)** Resting and IFN-γ activated BMDMs were infected with *M. abscessus* at an MOI of 10 and imaged using confocal microscopy 24 hours post-infection. Nuclei were visualized with DAPI, neutral lipids were stained with BODIPY 493/503, and mCherry-*M. abscessus was used* for infection visualization. Scale bars represent 10 μm. **(B)** Quantification of the average size of LDs per BMDM from **(A)** was conducted across 35 microscope fields. **(C)** Resting and IFN-γ activated BMDMs were infected with *M. abscessus* at an MOI of 10, with continuous IFN-γ stimulation, and bacterial load was detected 24 hours post-infection. **(D)** The concentration of IFN-γ in the cell culture supernatant from BMDMs was measured (*n* = 3). **(E)** Resting BMDMs and FAs-induced FMs were infected with *M. abscessus* at an MOI of 10 and imaged using confocal microscopy 24 hours post-infection. Nuclei were visualized with DAPI, neutral lipids were stained with BODIPY 493/503, and mCherry-*M. abscessus was used* for infection visualization. Scale bars represent 10 μm. **(F)** Quantification of the average size of LDs per BMDM from **(E)** was conducted across 40 microscope fields. **(G)** Resting BMDMs and FAs-induced FMs were infected with *M. abscessus* at an MOI of 10, and bacterial load was detected 24 hours post-infection (n=3). **(H)** IFN-γ administration and bacterial infection were conducted as per the schedule depicted in the diagram. WT mice were given 50ng IFN-γ intranasally one day prior to intratracheal infection, and treatment continued on days 1 and 3. Mice were infected intratracheally with *M. abscessus* at 5×10^7^ CFU and were euthanized on day 7. **(I)** Bacterial load in the lung lysate was determined on 7 dpi (*n* = 5). **(J)** Quantification of foam interstitial macrophages (FM-IMs) in lung tissue of mice by flow cytometry on 7 dpi (*n* = 5). **(K)** H&E and Oil Red O staining of lung sections from the model and IFN-γ group. Scale bars, 40 μm, 10 μm, respectively. Quantification of TAG **(L)** and T-CHO **(M)** in lung tissue of mice on 7 dpi (*n* = 5). Data are mean ± SEM and P values were determined by unpaired two-tailed Student’s t-test. Statistical significance was established at the levels of *p < 0.05, **p < 0.01 and ****p < 0.0001. MAB, *M. abscessus*.

Furthermore, the impact of IFN-γ supplementation on the formation of LDs in macrophage and the survival of *M. abscessus* was explored. LDs were stained with the BODIPY 493/503 and imaged using confocal microscopy 24h post-infection. For infection visualization, *M. abscessus* strains constitutively expressing mCherry were utilized. In BMDMs, neither IFN-γ treatment nor *M. abscessus* infection alone elicited LDs formation, whereas the combination of IFN-γ treatment and *M. abscessus* infection robustly induced the production of LDs (**Figures 3A-B**). Consistently, BMDMs pretreated with IFN-γ inhibited the intracellular survival of *M. abscessus* at 24 h post-infection (**Figure 3C**, **Supplementary Figure S3A**) and were characterized by a lower proportion of infected cells (**Supplementary Figure S3B**). Therefore, the addition of IFN-γ increases the formation of LDs and appears to restricts the growth of *M. abscessus in vitro* (5.91 Log_10_ decreased to 5.69 Log_10_). Concurrently, we quantified IFN-γ levels in cell supernatants. Infection with *M. abscessus* for 24h induced only marginal IFN-γ elevation in macrophages (pg/mL range), suggesting that exogenously supplemented IFN-γ synergizes with *M. abscessus* infection to drive the formation of LDs in BMDMs (**Figure 3D**). Previous studies have established a pivotal role for IFN-γ in controlling intracellular bacterial infection, while FMs has also been documented as a defense mechanism against *M.tb*. Therefore, to investigate the antimicrobial effects of FMs against *M. abscessus*, we generated FMs through exogenous fatty acid supplementation. Our results demonstrated that fatty acid-induced FMs reduced intracellular bacterial load (5.73 Log_10_ decreased to 5.51 Log_10_) (**Figures 3E-G**; **Supplementary Figure S3C**) and were characterized by a lower proportion of infected cells (**Supplementary Figure S3D**). Collectively, these findings suggest that LDs within FMs mediate IFN-γ-controlled containment of *M. abscessus* infection. Next, the effects of IFN-γ addition *in vivo* were investigated (**Figure 3H**). With intranasal treatment of IFN-γ prior to infection, bacterial counts in the lungs were reduced at 7 dpi (7.16 Log_10_ decreased to 6.87 Log_10_) (**Figure 3I**). By means of flowcytometry, it was shown that the proportion of FM-IMs in the lungs was significantly increased (**Figure 3J**). Additionally, histological analysis showed a significant reduction in the infiltration of inflammatory cells within the alveoli. Furthermore, Oil Red O staining demonstrated an increase in lipids content (**Figure 3K**). Subsequently, the levels of TAG and T-CHO in lung tissue were measured using colorimetry methods. In the IFN-γ treated group, the levels of TAG and T-CHO were significantly increased compared to the infected group (**Figures 3L-M**). These findings were consistent with the results of Oil Red O staining in lung tissue. In summary, the results demonstrated that IFN-γ supplementation increases the formation of FMs and restricts the growth of *M. abscessus*.

### 3.4 IFN-γ induced the formation of LDs that required CD40 activation

**Figure 4:**
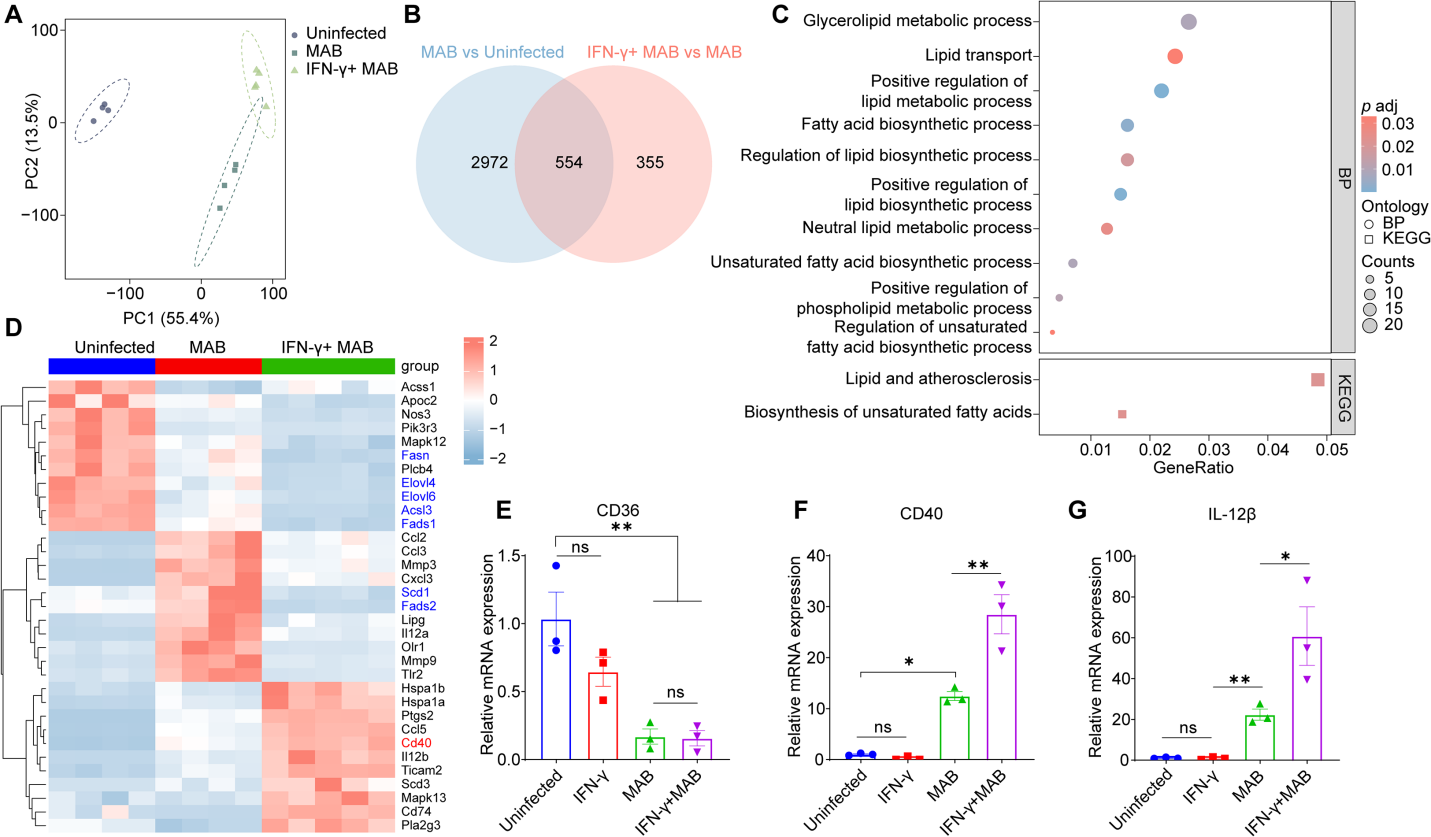
IFN-γ induced the formation of LDs that required CD40 activation. Transcriptome sequencing was performed on uninfected, MAB (*M. abscessus* infection) and IFN-γ+MAB (during *M. abscessus* infection with IFN-γ activation) groups. **(A)** Principal Component Analysis (PCA) for three groups. **(B)** Venn diagram of significantly regulated genes by MAB or by IFN-γ+MAB, as shown by transcriptome analysis (|Log_2_FC| >1; *P adj* < 0.05). **(C)** GO and KEGG enrichment analysis of the 554 differential genes involved in lipid metabolism (*P adj* < 0.05). **(D)** Heatmap of leading-edge genes in “fatty acid biosynthetic process, unsaturated fatty acid biosynthetic process and lipid and atherosclerosis” pathway. Genes encoding LDs synthesis are highlighted in blue, and the *CD40* gene is highlighted in red. **(E-G)** The mRNA expression of *CD36*, *CD40* and *IL-12β* in BMDMs. Data are mean ± SEM and *p* values were determined by one-way ANOVA with Dunnett’s multiple comparison tests. Statistical significance was established at the levels of *p < 0.05, **p < 0.01. MAB, *M. abscessus*.

Transcriptome sequencing was conducted to identify genes potentially responsible for the formation of a large number of LDs with a combination of IFN-γ pretreatment with *M. abscessus* infection (**Figure 4A**). A comparison of DEGs revealed that 554 genes overlapped in the uninfected group, the *M. abscessus* infection group and the *M. abscessus* infection group with IFN-γ activation (**Figure 4B**). Moreover, GO and KEGG enrichment analysis of these 554 DEGs indicated that FA biosynthetic process, unsaturated FA biosynthetic process, lipid and atherosclerosis were closely associated with IFN-γ treatment (**Figure 4C**). However, the heatmap of FA biosynthesis -related genes indicated that most LDs synthesis associated genes, such as *Elovl4*, *Elovl6*, *Fads1*, *Fads2*, *Fasn*, *Acsl3*, *Scd1*, *Fabp3*, were downregulated by IFN-γ treatment (**Figure 4D**). We discovered that the scavenger receptor CD36, a key regulator of lipid metabolism, was also downregulated in the context of *M. abscessus* infection or pretreatment with IFN-γ (**Figure 4E**). However, CD40, a costimulatory molecule, was strongly expressed during *M. abscessus* infection with IFN-γ activation (**Figure 4F**). Accordingly, qPCR validation revealed a significant upregulation of IL-12β, implying activation of CD40 signal upon IFN-γ treatment (**Figure 4G**).

### 3.5 IFN-γ-CD40-DGAT1 signal pathway activated LDs accumulation in *M. abscessus* infection

**Figure 5:**
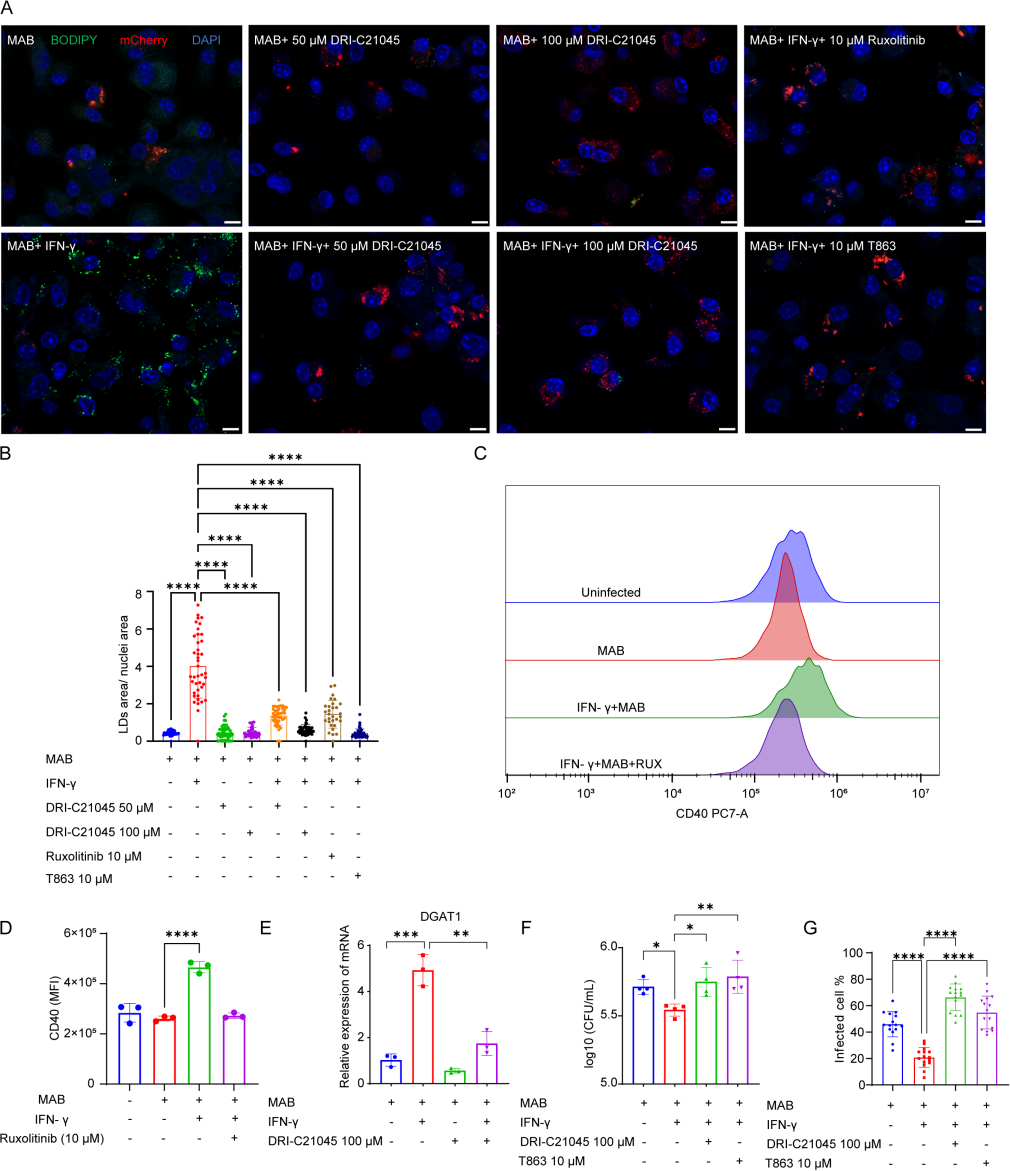
The IFN-γ-CD40-DGAT1 signaling pathway induces LDs accumulation during *M. abscessus* infection. **(A)** BMDMs activated by IFN-γ were infected with *M. abscessus*, and inhibitors of CD40 (DRI-C21045), DGAT1 (T863), and JAK1/2 (Ruxolitinib) were added after extracellular bacteria were cleared with amikacin. Cells were imaged using confocal microscopy 24 hours post-infection. Scale bars represent 10 μm. **(B)** Quantification of the average size of LDs per BMDM from **(A)** (*n* = 40 microscope fields). **(C, D)** Flow cytometry analysis of CD40 expression in *M. abscessus-*infected BMDMs treated with IFN-γ and the JAK1/2 inhibitor Ruxolitinib. **(E)** mRNA expression of *DGAT1* in *M. abscessus-*infected BMDMs treated with IFN-γ and the CD40 inhibitor DRI-C21045 (n=3). **(F)** Bacterial load in *M. abscessus-*infected BMDMs treated with IFN-γ, DRI-C21045 (CD40-CD40L costimulatory protein-protein interaction inhibitor), and T863 (DGAT1 inhibitor) at 24 hours post-infection (n=4). **(G)** Quantification of percentage of infected cells based on mCherry+ signal **(A)** was conducted across 15 microscope fields. Data are mean ± SEM and *p* values were determined by one-way ANOVA with Dunnett’s multiple comparison tests **(B, E, F)** or unpaired two-tailed Student’s t-test **(D)**. Statistical significance was established at the levels of *p < 0.05, **p < 0.01, ***p < 0.001, and ****p < 0.0001. MAB, *M. abscessus*.

Previous studies have shown that FGK45, an agonistic anti-CD40 monoclonal antibody, increases macrophages’ lipid uptake and lipid content (40). To determine if the formation of LDs under *M.abscessus* infection, with IFN-γ activation, was associated with CD40 activation, DRI-C21045, a potent and selective inhibitor of the CD40-CD40L interaction (38), was utilized to monitor changes in intracellular LDs content. It was observed that LDs were significantly inhibited in a concentration-dependent manner with DRI-C21045. Specifically, at a concentration of 100 μM DRI-C21045, the increase in LDs levels induced by IFN-γ activation was completely suppressed (**Figures 5A-B**). Furthermore, when a JAK1/2 inhibitor was applied, the lipid content of macrophages was also notably reduced (**Figures 5A-B**), suggesting that IFN-γ/JAK activation served as an upstream signal. Previous research has shown that IFN-γ-activated JAK1 modulates the cytokine profiles induced by CD40 in human antigen-presenting cells (41). Macrophages constitutively express CD40 at low levels, a process that is enhanced by IFN-γ (42). Indeed, the expression of CD40 is significantly upregulated during *M. abscessus* infection, particularly with IFN-γ activation. However, administering a JAK1/2 inhibitor can eliminate this phenomenon triggered by IFN-γ activation (**Figures 5C-D**). Therefore, IFN-γ activated JAK1 shifts CD40-induced accumulation of LDs. Functioning as the critical enzyme in TAG synthesis, DGAT1 mediates LDs formation in macrophages through its acyltransferase activity (43, 44). Then, we discovered that the accumulation of LDs was eliminated when macrophages were treated with T863, a chemical inhibitor of the DGAT1 enzyme (**Figures 5A-B**). In line with this, IFN-γ activation during *M. abscessus* infection also elevated the expression of key TAG biosynthesis genes, including *DGAT1*. Furthermore, the inhibition of CD40 signaling was found to significantly suppress the expression of *DGAT1*, as evidenced by RT-PCR (**Figure 5E**). Consequently, the IFN-γ-CD40-DGAT1 signaling pathway stimulates the accumulation of LDs. Although BMDMs pretreated with IFN-γ showed a significant inhibition of the intracellular survival of *M. abscessus*, the addition of DRI-C21045 and T863 negated this effect (**Figures 5F-G**). In summary, enhancing the formation of FMs reprogrammed by IFN-γ could counteract *M. abscessus* infection.

### 3.6 IFN-γ induced FA metabolism reprogramming in *M. abscessus*-infected BMDMs

**Figure 6:**
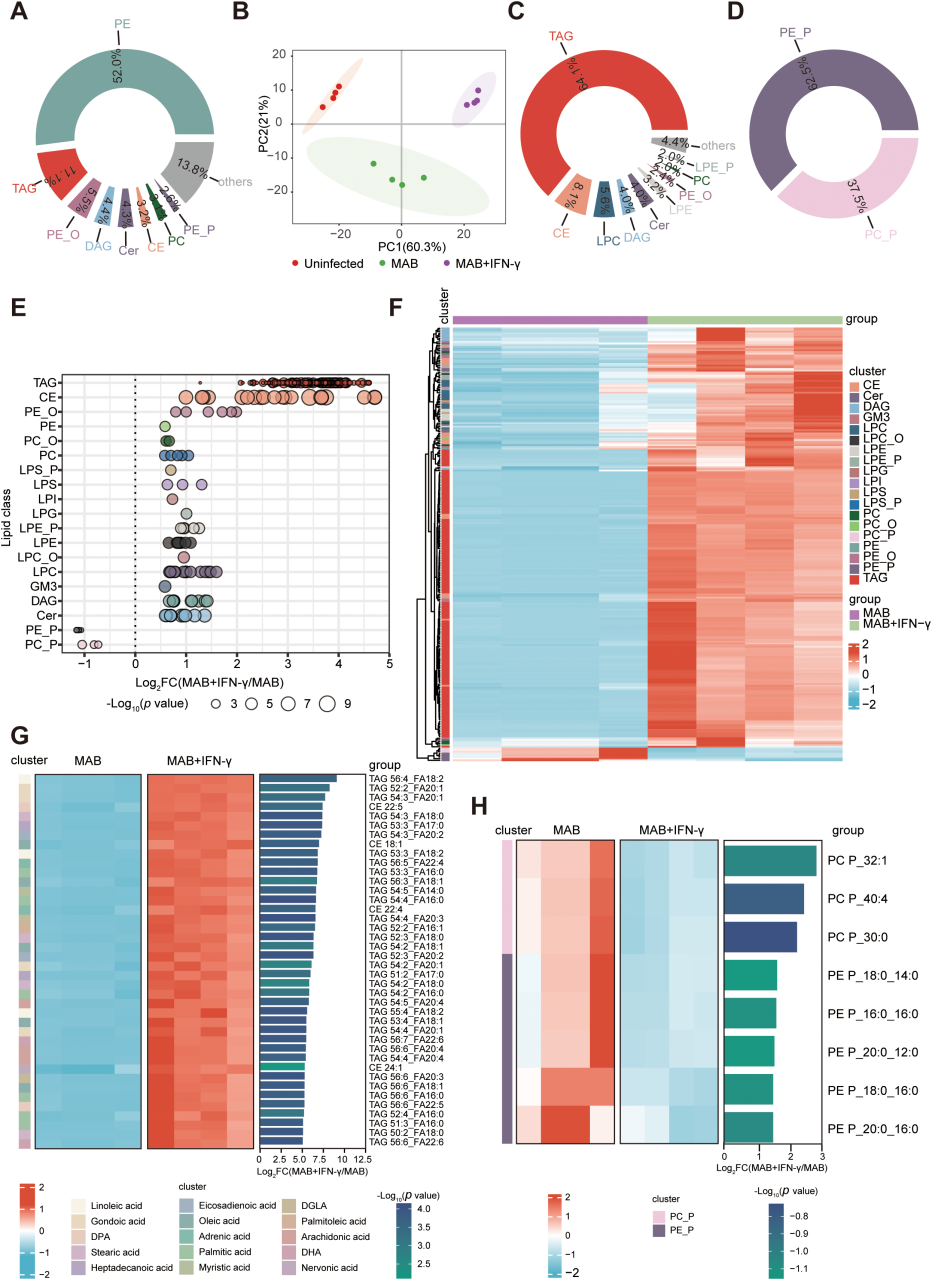
IFN-γ reprograms FA metabolism in *M. abscessus*-infected BMDMs. **(A)** Pie chart depicting the compositional distribution of identified lipid species across major classes in infected BMDMs. **(B)** PCA illustrating the clustering of Uninfected-BMDMs (Uninfected), *M. abscessus*-infected BMDMs (MAB), and *M. abscessus*-infected BMDMs treated with IFN-γ (MAB+IFN-γ) (n=4). **(C, D)** Donut plots quantifying the significantly upregulated or downregulated lipid species per class (Log_2_FC>0.58, *p* < 0.05). **(E)** Log_2_FC of lipid species summarized by lipid classes in MAB+IFN-γ versus MAB. Each bubble represents a lipid species, with its size indicating the *p* value. **(F)** Z-score heatmap of the differentially expressed lipid species in MAB+IFN-γ versus MAB. **(G)** The top 40 significantly elevated lipid species in MAB+IFN-γ compared to MAB. **(H)** All significantly reduced lipid species in MAB+IFN-γ compared to MAB. CE, Cholesteryl Ester; Cer, Ceramide; CL, Cardiolipin; DAG, Diacylglycerol; Gb3, Globotriaosylceramide; GM3, Monosialodihexosylganglioside; LacCer, Lactosylceramide; LPC, Lysophosphatidylcholine; LPC_O, Alkyl-lysophosphatidylcholine; LPE, Lysophosphatidylethanolamine; LPE_P, Plasmalogen-lysophosphatidylethanolamine; LPG, Lysophosphatidylglycerol; LPI, Lysophosphatidylinositol; LPS, Lysophosphatidylserine; LPS_P, Plasmalogen-lysophosphatidylserine; PC, Phosphatidylcholine; PC_O, Alkyl-phosphatidylcholine; PC_P, Plasmenyl-phosphatidylcholine; PE, Phosphatidylethanolamine; PE_O, Alkyl-phosphatidylethanolamine; PE_P, Plasmenyl-phosphatidylethanolamine; PS, Phosphatidylserine; MAB, *M. abscessus*.

**Figure 7:**
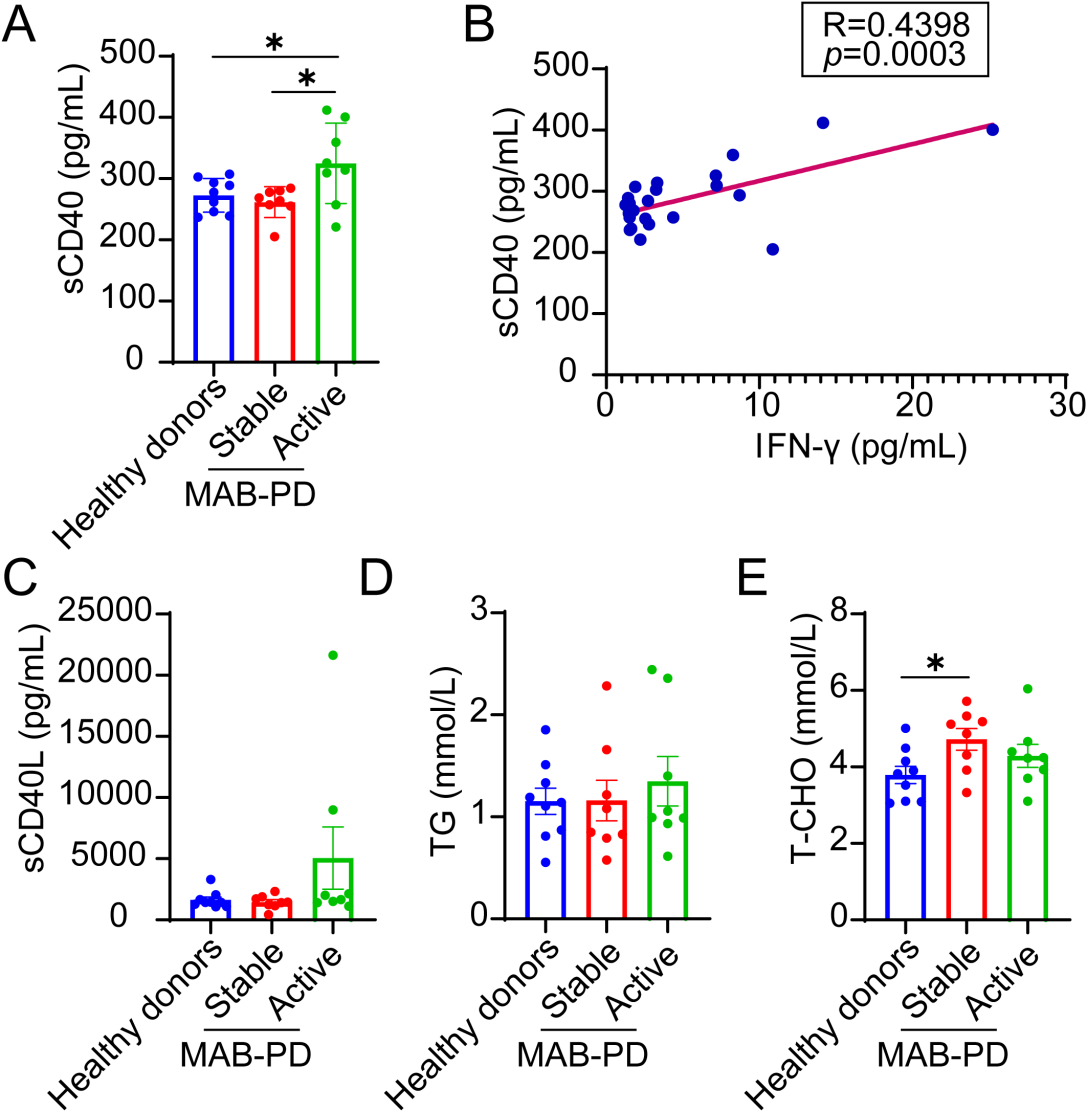
The correlation between serum IFN-γ and sCD40 levels in MAB-PD patients. **(A)** Serum sCD40 levels in healthy donors and in patients with stable or active MAB-PD. **(B)** Correlation between serum IFN-γ and sCD40 levels (R²=0.4398, *p* = 0.0003). **(C-E)** Serum sCD40L, TAG, and T-CHO levels in healthy donors and in patients with stable or active MAB-PD. Data are presented as mean ± SEM, and *p-*values were determined using an unpaired two-tailed Student’s t-test **(A, E)**. The Pearson correlation coefficient was used to investigate the relationship between serum IFN-γ and sCD40 levels **(B)**. Statistical significance was set at *p < 0.05. MAB-PD, *M.abscessus* pulmonary disease.

Building on our previous discovery of the IFN-γ-CD40-DGAT1 axis in FMs formation, we utilized lipidomics to delineate IFN-γ-driven lipid metabolic reprogramming in *M. abscessus*-infected BMDMs. Comparative analysis of lipid content in *M. abscessus*-infected macrophages with and without IFN-γ intervention revealed 35 significantly altered lipid species, predominantly phosphatidylethanolamine (PE) and TAG (**Figure 6A**). Principal component analysis (PCA) demonstrated global lipidomics remodeling in BMDMs co-stimulated with IFN-γ and *M. abscessus* infection, but not in those receiving IFN-γ alone (**Figure 6B**, **Supplementary Figure S4A**). Differential analysis revealed 256 dynamically regulated lipids (Log_2_FC > 0.58, *p* < 0.05), with 248 upregulated and 8 downregulated species (**Supplementary Figure S4B**). Targeted quantification showed a pronounced accumulation of intracellular TAG, diacylglycerol (DAG), and cholesteryl ester (CE), with TAG exhibiting the most robust induction. Conversely, phosphoethanolamine (PE_P) and phosphocholine (PC_P) were the predominant downregulated lipids (**Figures 7C-E**; **Supplementary Figure S4C**). Heatmap visualization further resolved the IFN-γ-modulated lipid signature in infected macrophages (**Figure 7F**). Strikingly, among the top 40 upregulated lipids ranked by *p*-value, only TAG and CE species were represented. Upregulated TAGs featured acyl chains including FA 18:2 (linoleic acid), FA 20:1 (eicosenoic acid), FA 18:1 (oleic acid), FA 16:0 (palmitic acid), and FA 20:4 (arachidonic acid) (**Figure 7G**). Integrated analysis identified metabolite cluster I (55 lipid species) as key IFN-γ-upregulated metabolites. Screening revealed that *M. abscessus* infection suppressed pro-inflammatory fatty acids, including FA 18:2 (linoleic acid) and FA 20:4 (arachidonic acid), while IFN-γ intervention counteracted this suppression and upregulated their levels (**Supplementary Figures S4B, D**). Downregulated lipids comprised membrane-associated phospholipids—primarily phosphatidylcholine (PC) and phosphatidylethanolamine (PE)—consistent with disrupted phospholipid metabolism and membrane dysfunction (**Figure 7H**).

### 3.7 A significant positive correlation exists between serum IFN-γ and sCD40 levels in MAB-PD

During mycobacterial infection, the IFN-γ-mediated upregulation of CD40 plays a crucial role in immunoregulation and also increases metalloproteinase activity. This leads to enhanced CD40 ectodomain cleavage and the subsequent release of soluble CD40 (sCD40) (45). To date, sCD40 has been identified as a new diagnostic and prognostic biomarker for certain diseases (46, 47). We subsequently measured the concentration of sCD40 in the serum of MAB-PD patients. Compared to healthy individuals and patients with stable disease, those with acute *M. abscessus* infection exhibited a significantly higher serum sCD40 concentration (**Figure 7A**). This mirrored the differences in serum IFN-γ concentration across the three groups. Furthermore, we analyzed the correlation between serum IFN-γ and sCD40, revealing a significant positive correlation (R^2^ = 0.4398, *p* = 0.0003) (**Figure 7B**). Concurrently, we also measured the serum levels of sCD40L, which had been implicated in the control of *M. tb* infection (27, 48), yet found no differences among the three groups (**Figure 7C**). We also examined the serum levels of TAG and T-CHO. Although no difference was noted in TAG levels, T-CHO levels were significantly higher in stable patients compared to healthy individuals (**Figures 7D-E**). No correlation was observed between elevated IFN-γ and TAG levels in the serum, which may necessitate further investigation using mononuclear-derived macrophages.

## 4 Discussion

**Figure 8:**
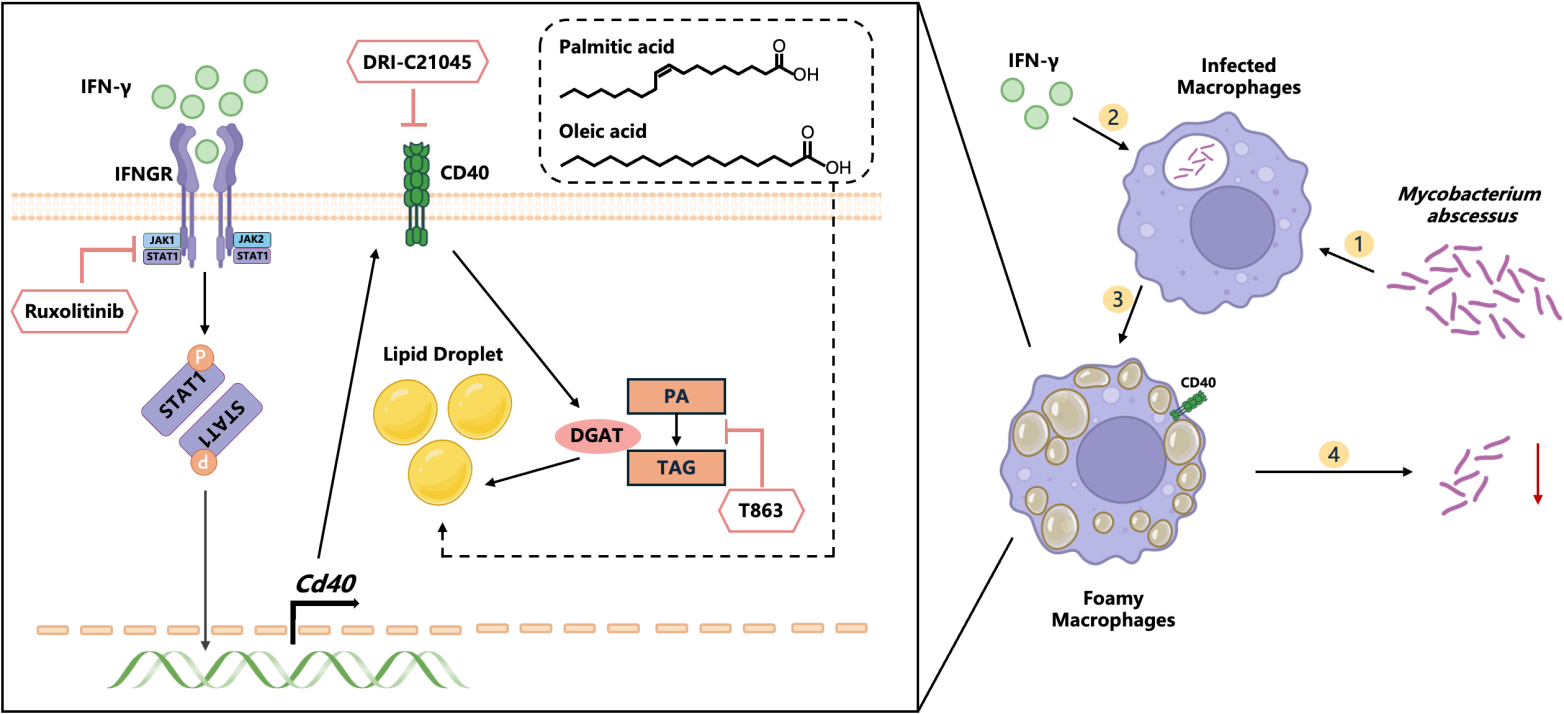
Model illustrating how IFN-γ induces the formation of FMs through CD40-DGAT1 signaling to control *M. abscessus* pulmonary infection. IFN-γ activates JAK1, which shifts CD40-induced metabolic reprogramming profiles, leading to the accumulation of LDs. The increased formation of FMs reprogrammed by IFN-γ significantly inhibits the intracellular survival of *M. abscessus*. Moreover, FMs induced by free fatty acids (oleic acid and palmitic acid) also enhance the clearance of *M. abscessus*. In summary, the addition of IFN-γ represents a potential pathway to rescue *M. abscessus* infection.

To date, the mechanisms underlying the formation FMs in *M. abscessus-*induced lung infections remain unclear. Our current data demonstrated that IFN-γ induced CD40 signal activation plays an important role in both the formation of FMs and the control of *M. abscessus* (**Figure 8**). The relationship between *Mycobacterium* infection and host lipids remains a challenging enigma. *M.tb* exploits WNT6/ACC2-induced storage of TAG in macrophages to facilitate its intracellular survival (49). FMs, induced by lepromatous leprosy, actively contribute to the increased survival of *M. leprae* within the host (50). However, recent studies question this principle by indicating that in *M.tb*-infected macrophages, the formation of LDs prevents bacterial acquisition of host FAs while supporting the production of FAs-derived protective lipid mediators (51). Lipid loading of RAW 264.7 cells and mouse peritoneal macrophages with either oxidized or acetylated LDL significantly inhibits the growth of *Chlamydia pneumoniae* (*C. pneumoniae*) (52). In our study, we found that monocytes in patients with active *M.abscessus* infection contained notably more LDs. Infected WT mice exhibited a robust anti- *M.abscessus* effect, which was accompanied by the formation of granulomatous lesions and lipids. Moreover, the addition of IFN-γ increases the formation of FMs and restricts the growth of *M. abscessus* both *in vitro* and *in vivo*.

During infection, host cytokines contribute to the formation of FMs. The Bacillus Calmette-Guérin (BCG)-induced LDs formation is TLR2 mediated and involves inducible intracellular domains that may function as signaling platforms in the production of inflammatory mediators (53). The induction of macrophages by M-CSF results in enhanced FMs formation via CD36 during monocyte-to-macrophage differentiation (54). *C. pneumoniae*-induced extracellular IL-1β triggers a negative feedback loop that inhibits GPR109a and ABCA1 expression, as well as cholesterol efflux, leading to the accumulation of intracellular cholesterol and the formation of FMs (55). Additionally, *in vitro* studies suggested that IL-17A activates vascular endothelial cells, which secrete cytokines that in turn enhance FMs formation in macrophages (56). During *M. tb* infection, bacterial UreC protein triggers host genomic damage and cGAS-STING-dependent IFN-β production, which facilitates FMs formation via SR-A1, creating a lipid-rich niche that enhances bacterial survival and impairs immunity (57). Under treatment for tuberculous pleural effusions, IL-10 was found to promote the accumulation of LDs in macrophages through cytokine depletion. IL-10 deficiency partially prevented the induction of the foamy phenotype (58). Meanwhile, Kajiwara et al. discovered that AIM fosters an immunosuppressive environment, primarily driven by the production of IL-10 and the associated polarization of M2 macrophages. This specific immune landscape promotes the formation of FMs and facilitates the persistence of *M. avium* infection (36, 59). In addition, Knight et al. identified an IFN-γ/HIF-1α/Hig2 pathway that was operative both *in vitro* in primary macrophages, and in an *in vivo* aerosol model of *M. tb* infection (18). Our findings also demonstrated that IFN-γ plays a significant role in the formation of FMs induced by *M. abscessus*. Elevated levels of serum IFN-γ were detected in acute MAB-PD patients and in the lung tissue of infected mice. Furthermore, lipid aggregation was not observed in the lungs of IFN-γ^-/-^ infected mice. Consequently, IFN-γ was found to be required for the formation of FMs in *M. abscessus* infection.

While the biogenesis of FMs has been thoroughly investigated in the context of atherosclerosis (55, 60), the mechanisms underlying intracellular lipid accumulation during *mycobacterial* infection remain poorly understood. In contrast to FMs in atherosclerosis, which engulf large amounts of oxidized low-density lipoprotein (oxLDL) and are enriched in cholesterol (61), FMs within tuberculous pulmonary lesions primarily accumulate TAGs. The lipid microenvironment constructed by very low-density lipoprotein actually facilitates the persistence and latency of *M. avium* (62). Moreover, high levels of oxLDL in patients with type 2 diabetes mellitus impair the lysosomal function of macrophages, leading to bacterial persistence (63). As a major and distinguishing cell wall biolipid from *M.tb*, mycolic acid was found to transactivate TLR4 (64) and interferes with the lipid homeostasis of alveolar macrophages, thereby inducing the differentiation into FMs, thus exhibiting an increased proinflammatory function (65). In our studies, supplementation with FAs or IFN-γ-induced FMs formation both demonstrated a significant dampening effect on the intracellular bacterial burden. In summary, different fatty acid signals may trigger different metabolic and functional states in macrophages, suggesting that lipid particles may have a dual role during the infection process.

More notably, M1 macrophages stimulated with LPS and IFN-γ exhibit substantial intracellular LDs accumulation, whereas M0 or M2 macrophages show minimal accumulation. This suggests that LDs accumulation may serve as a structural biomarker for macrophage phenotype (66). Concurrently, LDs biogenesis requires combined LPS and IFN-γ stimulation, which suppresses fatty acid oxidation and drives DGAT1-dependent TAG synthesis. Critically, the pro-inflammatory function of LDs depends on their facilitation of PGE2-mediated secretion of pro-inflammatory cytokines (67). Our lipidomics analysis revealed that IFN-γ-induced FMs predominantly upregulate TAGs enriched with diverse fatty acid chains, among which linoleic acid was the most prominently enriched fatty acid. Current evidence indicates that linoleic acid metabolism plays a pivotal immunomodulatory role during microbial infections. During BCG infection, linoleic acid enhances trained immunity by promoting the secretion of pro-inflammatory cytokines such as TNF-α, IL-6, and IL-1β (68). Similarly, in *Brucella* infection models, linoleic acid restricts intracellular bacterial burden via enhanced nitrite accumulation and augments levels of IL-12 and IFN-γ in infected mice (69).

IFN-γ therapy acts as an effective immunomodulatory adjuvant for tuberculosis, especially for multidrug-resistant (MDR-TB) and severe cases. It enhances the host’s immune response by activating macrophages, improving treatment outcomes when used in conjunction with standard antibiotics. It accelerates sputum conversion, promotes lung cavity healing, and improves radiological and immunological markers (70). Exogenous IFN-γ treatment now has been proven to be capable of successfully curing refractory *M. avium* infection (71). Hence, host immunity mediated by IFN-γ may plays a critical role in controlling NTM infections. While, in patients with persistent NTM infection, specific distribution characteristics of B-cell and T-cell subsets are closely associated with the presence of Anti-IFN-γ autoantibody (AIGA) (72). Patients with AIGA exhibit impaired IFN-γ signaling, leading to severe disseminated intracellular pathogen infections, particularly involving NTM (73). In severe NTM disease, antimycobacterial therapy alone is often ineffective in the absence of intact IFN-γ signaling, as evidenced by the extremely poor survival of patients with complete IFN-γ receptor deficiency. In contrast, patients with partial defects in IFN-γ signaling demonstrate better survival, indicating that even minimal IFN-γ signaling can effectively control mycobacterial infection (74). Currently, the immunotherapy for AIGA, including rituximab or cyclophosphamide, and it has shown effectiveness in some cases (75). Anti-CD20 therapy (Rituximab) works by inhibiting the differentiation of new, pathogenic, autoantibody-producing plasma cells. After the patient received immunotherapy, they showed signs of infection clearance or alleviation, inflammation subsidence, decreased levels of autoantibodies against IFN-γ, and improved IFN-γ signal transduction (76–78).

In our study, we discovered that IFN-γ-activated JAK1 alters the accumulation of LDs induced by CD40, and the formation of LDs is significantly reduced when the CD40-DGAT1 signal is blocked. Correspondingly, CD40-knockout mice exhibit a significantly reduced atherosclerotic plaque area and diminished inflammatory burden (26). Previous studies have demonstrated that the CD40 signal stimulates unconventional metabolic reprogramming to promote the expression of pro-inflammatory genes and anti-tumorigenic phenotypes in macrophages (40). During *M. tb* infection, CD40 synergizes with TLR-4 to enhance dendritic cell maturation and induce autophagic clearance of intracellular bacteria (79).

In summary, our results suggest that IFN-γ supplementation could represent a novel therapeutic strategy for treating *M. abscessus* pulmonary infections. This study is the first to identify the IFN-γ-CD40-DGAT1 axis as a crucial regulator in the formation of FMs and the control of *M. abscessus* infection. Future investigations will explore the potential of sCD40 and FMs as prognostic biomarkers in MAB-PD, as well as the therapeutic potential of linoleic acid in managing *M. abscessus* infection.

## Data Availability

Original contributions presented in this study are included in the article/**Supplementary Material**. The sequencing dataset generated from this study has been deposited in the NCBI database under the GEO accession number GSE287681 (https://www.ncbi.nlm.nih.gov/geo/query/acc.cgi?acc=GSE287681). Further inquiries can be directed to the corresponding author.

## Supplementary material

Supplementary Figure 1 Gating strategy for human-PBMCs. Cell counts for human PBMCs were conducted and finally 2x10^6 cells were utilized for staining. During flow cytometry acquisition, 1×10^6 cells were collected from the “all events” population. The MFI of BODIPY in Dye^-^CD14^+^ cell population were measured.

Supplementary Figure 2 Rag2^-/-^ mice did not exhibit granulomatous lesions or LDs formation upon *M. abscessus* infection. Rag2^-/-^ mice were intratracheally infected with cultured *M. abscessus* at a concentration of 5×10^7^ CFU and were euthanized on days 7, 21, and 35 post-infection (dpi). (A) Micrographs of hematoxylin and eosin-stained lung sections at 0 dpi and on 7, 21, and 35 dpi. Scale bars, 20 μm. (B) Neutral lipids staining of lung cryosections with Oil Red O at 0 dpi and on 7, 21, and 35 dpi. Scale bars, 10 μm. Abbreviations: MAB, *M. abscessus*.

Supplementary Figure 3 Reprogramming of lipid metabolism in *M. abscessus*-infected BMDMs. (A) Quantification of the average size of LDs per mCherry+ BMDM from (**Figure 3A**) was conducted across 40 cells. (B) Quantification of percentage of infected cells based on mCherry+ signal (**Figure 3A**) was conducted across 15 microscope fields. (C) Quantification of the average size of LDs per mCherry+ BMDM from (**Figure 3E**) was conducted across 40 cells. (D) Quantification of percentage of infected cells based on mCherry+ signal (**Figure 3A**) was conducted across 15 microscope fields.

Supplementary Figure 4 Reprogramming of lipid metabolism in *M. abscessus*-infected BMDMs. (A) A PCA displaying the clustering of Uninfected, Uninfected+IFN-γ, MAB, MAB+IFN-γ, and Quality Control (QC) groups ((n=4). (B) The distributions of significantly altered lipid species between two groups (“MAB vs Uninfected” and “MAB+IFN-γ vs MAB”) and the lipid species that showed reversed significance. (C) The top 9 significantly elevated lipid species in MAB+IFN-γ vs MAB, presented as a Violin plot. (D) Log_2_FC of lipid species, categorized by lipid classes, in the lipid species that showed reversed significance. Abbreviations: MAB, *M. abscessus*.
