# Supplementary figures and images for: IFN-γ induced the formation of foamy macrophages via CD40 signal to control Mycobacterium abscessus pulmonary infection
Source: Front Immunol. 2025 Nov 27;16:1697443. doi: 10.3389/fimmu.2025.1697443 (PMC12695758; doi:10.3389/fimmu.2025.1697443)

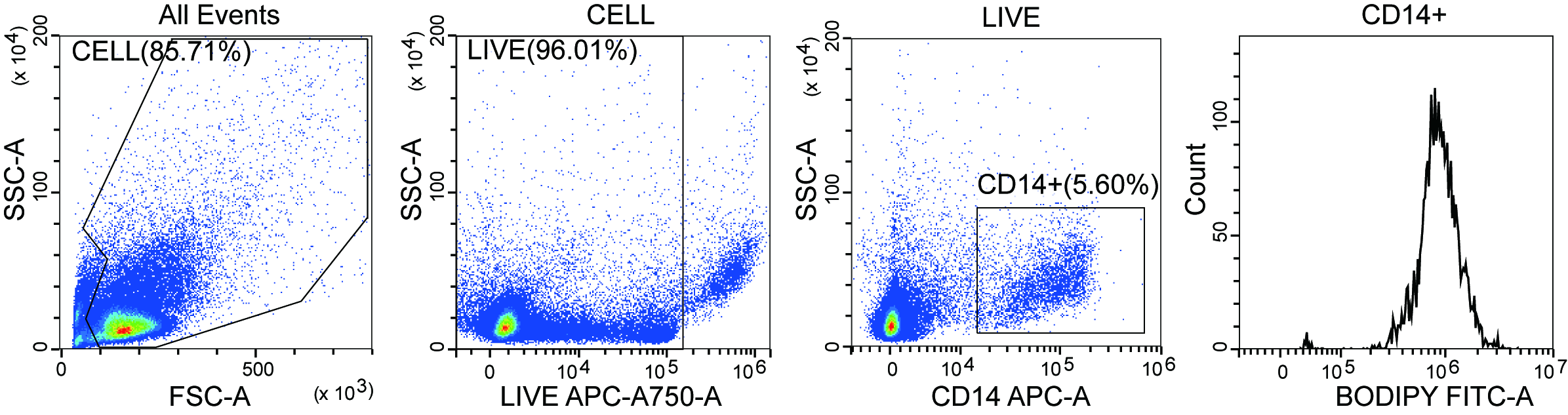

Supplement: Supplementary Figure 1 — Gating strategy for human-PBMCs. Cell counts for human PBMCs were conducted and finally 2x10^6 cells were utilized for staining. During flow cytometry acquisition, 1×10^6 cells were collected from the “all events” population. The MFI of BODIPY in Dye-CD14+ cell population were measured. [file Image1.tif]

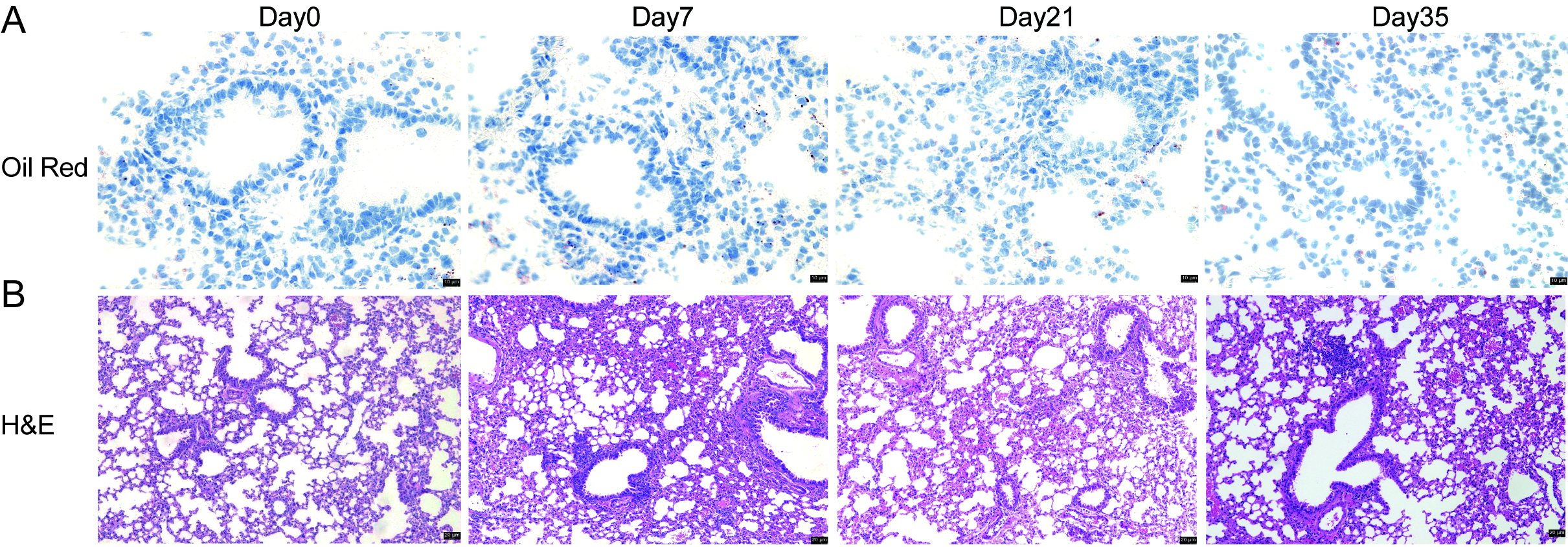

Supplement: Supplementary Figure 2 — Rag2-/- mice did not exhibit granulomatous lesions or LDs formation upon M. abscessus infection. Rag2-/- mice were intratracheally infected with cultured M. abscessus at a concentration of 5×10^7^ CFU and were euthanized on days 7, 21, and 35 post-infection (dpi). (A) Micrographs of hematoxylin and eosin-stained lung sections at 0 dpi and on 7, 21, and 35 dpi. Scale bars, 20 μm. (B) Neutral lipids staining of lung cryosections with Oil Red O at 0 dpi and on 7, 21, and 35 dpi. Scale bars, 10 μm. Abbreviations: MAB, M. abscessus. [file Image2.tif]

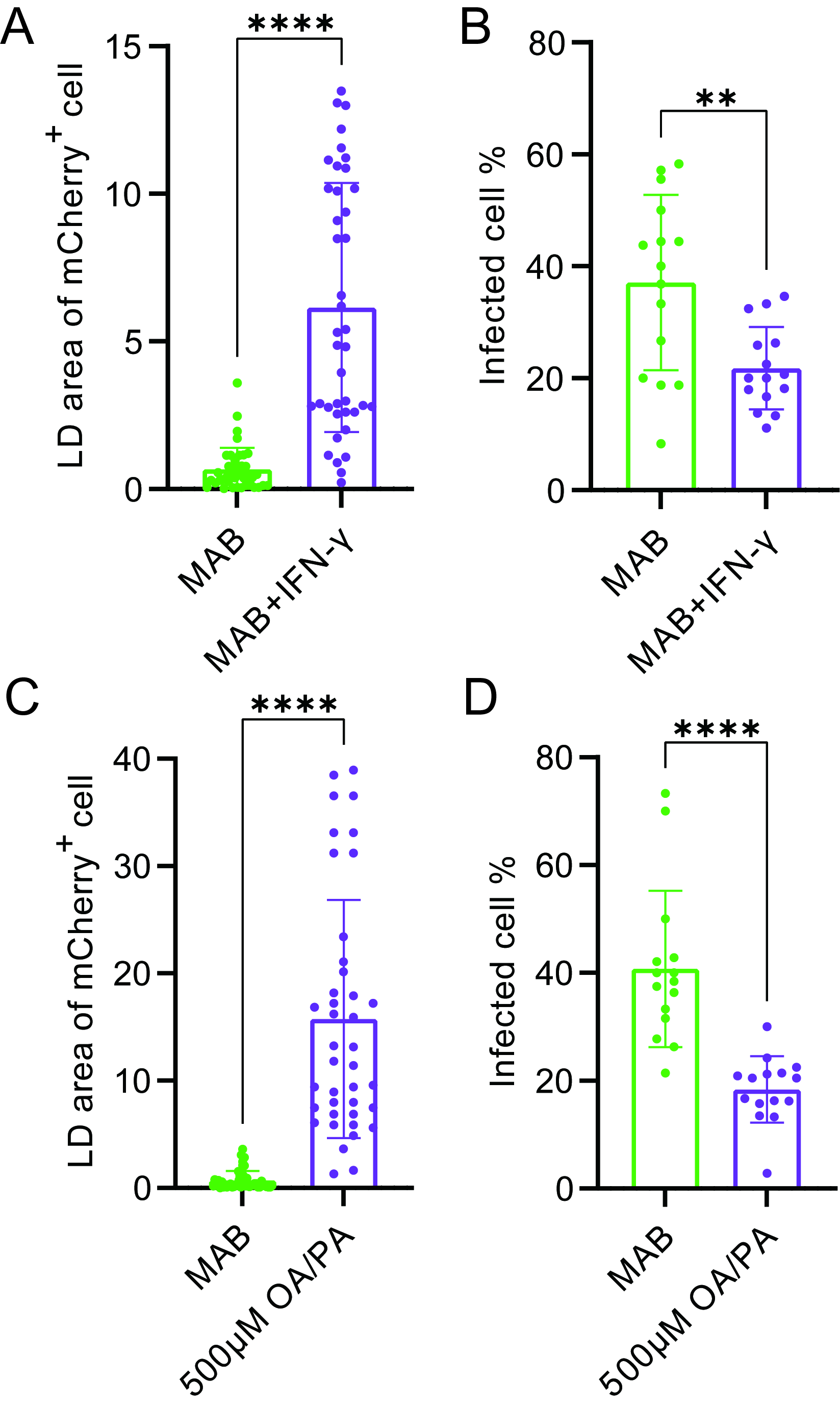

Supplement: Supplementary Figure 3 — Reprogramming of lipid metabolism in M. abscessus-infected BMDMs. (A) Quantification of the average size of LDs per mCherry+ BMDM from (Figure 3A) was conducted across 40 cells. (B) Quantification of percentage of infected cells based on mCherry+ signal (Figure 3A) was conducted across 15 microscope fields. (C) Quantification of the average size of LDs per mCherry+ BMDM from (Figure 3E) was conducted across 40 cells. (D) Quantification of percentage of infected cells based on mCherry+ signal (Figure 3A) was conducted across 15 microscope fields. [file Image3.tif]

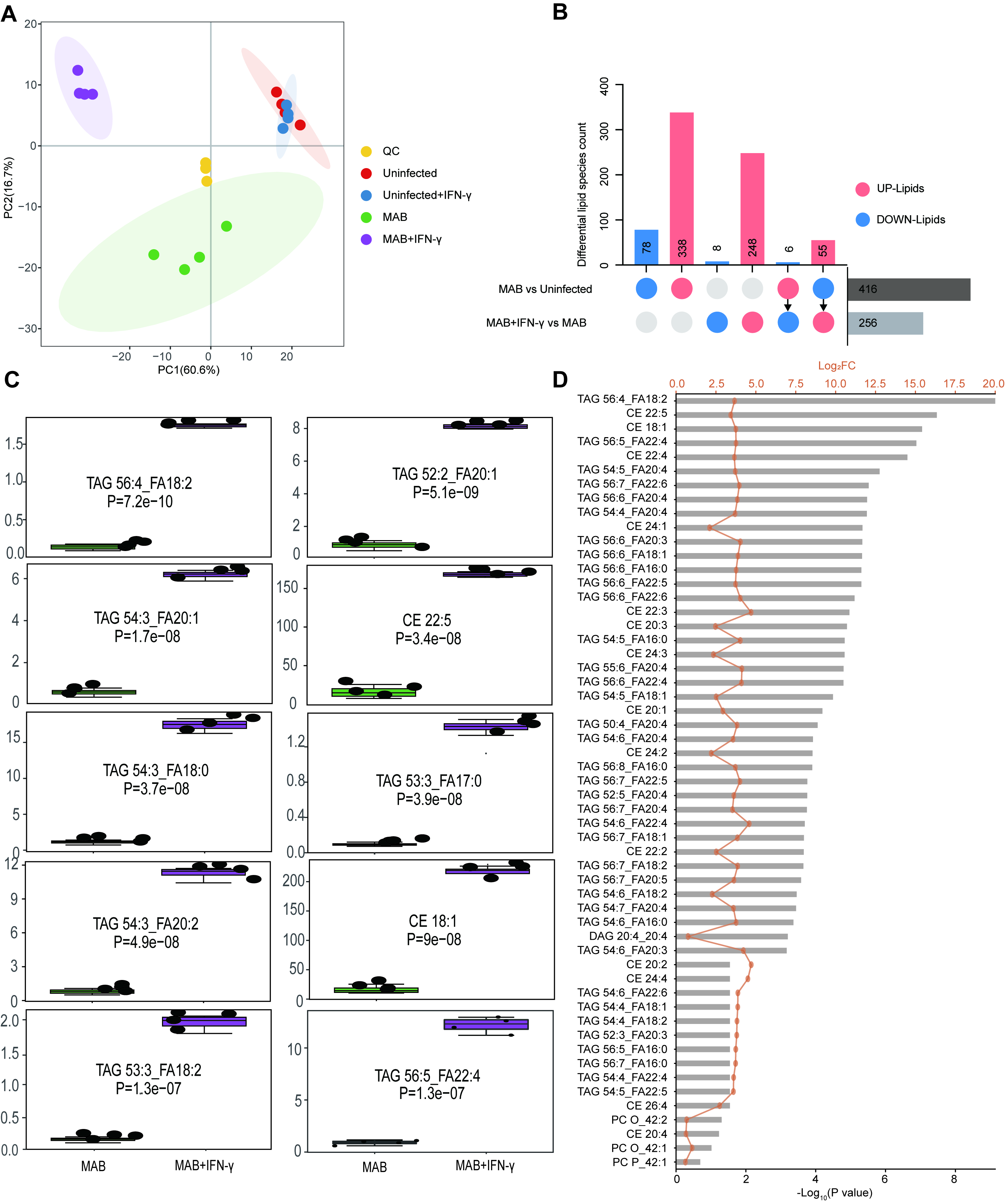

Supplement: Supplementary Figure 4 — Reprogramming of lipid metabolism in M. abscessus-infected BMDMs. (A) A PCA displaying the clustering of Uninfected, Uninfected+IFN-γ, MAB, MAB+IFN-γ, and Quality Control (QC) groups ((n=4). (B) The distributions of significantly altered lipid species between two groups (“MAB vs Uninfected” and “MAB+IFN-γ vs MAB”) and the lipid species that showed reversed significance. (C) The top 9 significantly elevated lipid species in MAB+IFN-γ vs MAB, presented as a Violin plot. (D) Log2FC of lipid species, categorized by lipid classes, in the lipid species that showed reversed significance. Abbreviations: MAB, M. abscessus. [file Image4.tif]
